# Lipofuscin Granule Bisretinoid Oxidation in the Human Retinal Pigment Epithelium forms Cytotoxic Carbonyls

**DOI:** 10.3390/ijms23010222

**Published:** 2021-12-25

**Authors:** Marina Yakovleva, Alexander Dontsov, Natalia Trofimova, Natalia Sakina, Alexey Kononikhin, Arseny Aybush, Alexander Gulin, Tatiana Feldman, Mikhail Ostrovsky

**Affiliations:** 1 Emanuel Institute of Biochemical Physics, Russian Academy of Sciences, Kosygin st. 4, 119334 Moscow, Russia; lina.invers@gmail.com (M.Y.); adontsovnick@yahoo.com (A.D.); ntrofimova@mail.ru (N.T.); nsakina@mail.ru (N.S.); alex.kononikhin@gmail.com (A.K.); ostrosky3535@mail.ru (M.O.); 2 Skolkovo Institute of Science and Technology, Bolshoy Boulevard 30, bld. 1, 121205 Moscow, Russia; 3 N.N. Semenov Federal Research Center for Chemical Physics, Russian Academy of Sciences, Kosygin st. 4, bld. 1, 119991 Moscow, Russia; aiboosh@gmail.com (A.A.); aleksandr.gulin@phystech.edu (A.G.); 4 Department of Biology, Lomonosov Moscow State University, Leninskiye Gory 1, 119234 Moscow, Russia

**Keywords:** age-related macular degeneration, retinal pigment epithelium, lipofuscin granules, bisretinoid fluorophores, bisretinoid oxidation products, cytotoxic carbonyls, hydrophilicity, amphiphilicity

## Abstract

Age-related macular degeneration (AMD) is the primary cause of central blindness among the elderly. AMD is associated with progressive accumulation of lipofuscin granules in retinal pigment epithelium (RPE) cells. Lipofuscin contains bisretinoid fluorophores, which are photosensitizers and are phototoxic to RPE and neuroretinal cells. In the presence of oxygen, bisretinoids are also oxidized, forming various products, consisting primarily of aldehydes and ketones, which are also potentially cytotoxic. In a prior study, we identified that in AMD, bisretinoid oxidation products are increased in RPE lipofuscin granules. The purpose of the present study was to determine if these products were toxic to cellular structures. The physicochemical characteristics of bisretinoid oxidation products in lipofuscin, which were obtained from healthy donor eyes, were studied. Raman spectroscopy and time-of-flight secondary ion mass spectrometry (ToF–SIMS) analysis identified the presence of free-state aldehydes and ketones within the lipofuscin granules. Together, fluorescence spectroscopy, high-performance liquid chromatography, and mass spectrometry revealed that bisretinoid oxidation products have both hydrophilic and amphiphilic properties, allowing their diffusion through lipofuscin granule membrane into the RPE cell cytoplasm. These products contain cytotoxic carbonyls, which can modify cellular proteins and lipids. Therefore, bisretinoid oxidation products are a likely aggravating factor in the pathogenesis of AMD.

## 1. Introduction

Age-related macular degeneration (AMD) is a chronic eye disease characterized by damage to the macular region and progressive central vision loss [1]. AMD is a complex disease with multiple risk factors and disease pathologies [2]. Oxidative stress is central to the development of AMD [2,3,4,5]. It is characterized by increased levels of reactive oxygen species (ROS) resulting in damage or modification of cellular proteins, lipids, and DNA, impairing their physiological functions [5]. Lipofuscin granules (LGs) are one of the sources of ROS in retinal pigment epithelium (RPE) cells. Light exposure induces ROS formation in LGs, initiating oxidative stress in RPE cells [6,7]. Therefore, development of AMD is associated with progressive accumulation of LGs in the RPE [8]. LGs are therefore a risk factor for degenerative processes in the retina and RPE [9,10,11,12,13].

LGs accumulate in RPE cells with age [14,15,16]. This process is increased in several retinal degenerative diseases, especially AMD [17,18,19]. LGs are formed by incomplete lysosomal degradation of photoreceptor outer segment (POS) debris following phagocytosis of shed POSs by RPE cells [20,21]. LGs are considered membrane-bound residual bodies of the lysosomal compartment of the RPE cell [8]. They contain bisretinoids fluorophores (BisRets), which are byproducts of all-*trans* retinal modifications [22]. *N*-retinyl-*N*-retinylidene ethanolamine (A2E) is the most widely studied BisRet [23,24,25]. BisRets and their derivatives are major sources of LG fluorescence [8,9].

The features of BisRets, such as photosensitizers, have been studied in detail [6,7,26,27,28]. However, in the presence of oxygen, BisRets themselves can be photo-oxidized to form various products, consisting primarily of epoxides, peroxides, aldehydes, and ketones, which are potentially cytotoxic [29,30,31,32,33,34,35]. Importantly, the processes of BisRet oxidation in LGs by non-lipofuscin ROS in the absence of light has not been fully investigated. For example, mitochondrially generated ROS in RPE cells [36] can lead to BisRet oxidation and degradation. This could be exacerbated by mitochondrial damage in the RPE, which contributes to the pathogenesis of AMD [2,37]. 

The cytotoxic properties of photooxidation and photodegradation products (BisRet-OX) in LGs have not been fully investigated. The role of these compounds in pathological processes of the RPE remains controversial. Some studies have suggested that highly reactive cytotoxic carbonyl compounds, aldehydes and ketones, are formed during photo-oxidation of BisRets in LGs [38,39,40]. By contrast, other studies [41,42] suggest BisRet-OX; interacts with itself or with A2E, forming products with a higher molecular weight inside LGs. Most of these compounds are also hydrophobic and remain inside LGs, resulting in the concomitant diminution of its reactivity in vivo [41,42].

Clarifying the roles of BisRet-OX is important to delineate the mechanisms of pathological ocular diseases, especially AMD. Our prior findings have demonstrated that LG BisRet-OX content is higher in AMD eyes than in normal eyes, which was indicated by changes in the characteristics of LG fluorescence spectra and in the parameters of fluorescence decay kinetic curves [9]. Specifically, the fluorescence excitation at 488 nm of samples from eyes with AMD increases the fluorescence intensity of the band at 556 nm, and the contribution of BisRet-OX to total fluorescence increases. However, the pathophysiological or protective properties of these products remain controversial, as prior studies have suggested conflicting roles [41,42], and as BisRet-OX could also potentially become a neutral product eventually. Investigating the potential release of BisRet-OX from LGs into the RPE cell cytoplasm and the assessment of their toxicity to cellular structures is thus fundamental to understanding the pathogenesis of retinal diseases.

The present study aimed to characterize the physicochemical characteristics of BisRet-OX products and their potential release from LGs into the RPE cell cytoplasm. To obtain BisRet-OX, two LG oxidation models were used, including irradiating LGs with visible light (photo-oxidative destruction of BisRets), and exposing LGs to superoxide radicals (oxidative destruction of BisRets).

## 2. Results

### 2.1. Effect of Light and Superoxide Radicals on the Fluorescent Properties of Lipofuscin Granules (LGs)

The composition of LG fluorophores from human RPE cells includes approximately 20 BisRets and other all-*trans* retinal derivatives, which undergo photo-oxidative destruction under light exposure [31,43,44]. BisRet-OXs with increased hydrophilicity were previously thought to leave LGs and enter RPE cell cytoplasm [34], directly damaging cell structures [45].

In the present study, BisRet-OX formation was evaluated by measuring the change in the fluorescence spectra of samples before and after irradiation with visible light, as well as after exposure to superoxide radicals. During BisRet oxidation, the absorption and fluorescence spectra of fluorophores shifted to the short-wave region [31,46]. We used buffer solution with LGs (subsequently referred to as LG suspension), the sediment from the LG suspension (subsequently referred to as LG sediment), and the supernatant (subsequently referred to as supernatant).

Three wavelengths of 340, 365, and 488 nm were used to excite fluorescence. The 488 nm wavelength is traditionally used clinically to assess fundus autofluorescence [47]. Two other wavelengths (340 and 365 nm) were used for a more complete study of the spectral properties of LG fluorophores, most of which absorb in the short-wave region, especially the oxidation products.

**Figure 1:**
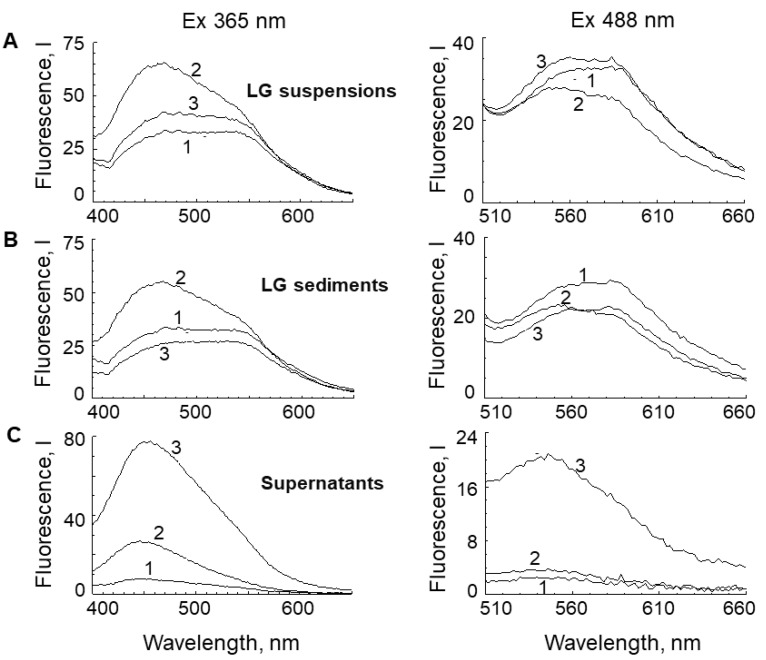
Fluorescence spectra of lipofuscin granule (LG) suspensions (**A**), LG sediments (**B**), and supernatants (**C**). 1—control: LG suspension before light irradiation or incubation with superoxide radicals; sediment and supernatant obtained from this suspension. 2—LG suspension after 60 min visible light irradiation; sediment and supernatant obtained from this suspension. 3—LG suspension after 60 min incubation with potassium superoxide; sediment and supernatant obtained from this suspension. The wavelengths of fluorescence excitation were 365 nm (left) and 488 nm (right).

Irradiation of the LG suspension with visible light or exposure to superoxide radicals caused increased fluorescence intensity in the short-wave region of 450–550 nm, which indicated increased BisRet-OX contents [31,46] The spectral changes under different LG fluorophore oxidation conditions varied (Figure 1).

Importantly, in the supernatant obtained from the original LG suspension, the fluorescence intensity was very low, indicating a low content of hydrophilic oxidized forms of LG fluorophores (Figure 1C, spectra 1 at fluorescence excitations of 365 and 488 nm). However, after oxidation of the LG suspension, the fluorescence intensity of the supernatants increased sharply in the short-wavelength region of the spectrum (450 nm, Ex. 365 nm; 550 nm, Ex. 488 nm). This was particularly apparent in LG suspension exposed to superoxide radicals (Figure 1C, spectra 3, fluorescence excitations 365 and 488 nm). 

This suggested that when the LG suspension was irradiated with visible light or exposed to superoxide radicals, BisRet-OXs were formed, some of which had hydrophilic properties and could potentially diffuse from LGs into RPE cell cytoplasm. 

**Figure 2:**
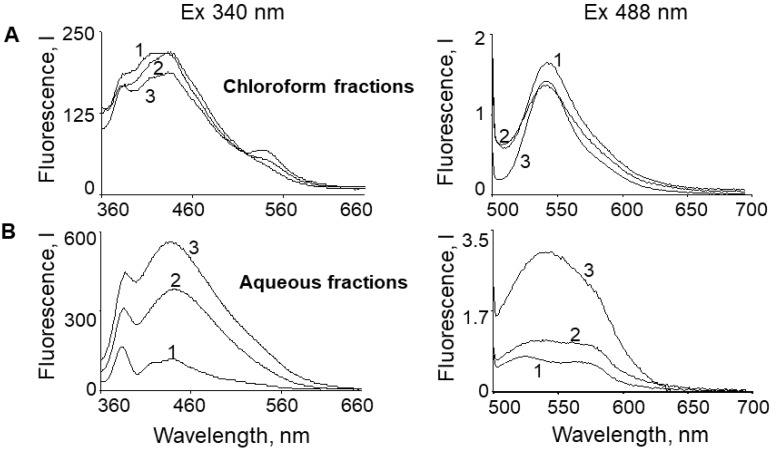
Fluorescence spectra of chloroform (**A**) and aqueous (**B**) fractions of the supernatants during extraction. 1—control: supernatant fractions from the original (control) lipofuscin granule (LG) suspension, 2—supernatant fractions from LG suspension irradiated for 30 min, and 3—supernatant fractions from LG suspension oxidized with superoxide. The wavelengths of fluorescence excitation were 340 nm (left) and 488 nm (right).

Not only hydrophilic compounds, but also substances with amphiphilic properties, can pass into the aqueous phase. Therefore, to study the nature of fluorophores diffusing into aqueous medium in more detail, chloroform was added to the supernatants (Figure 1C). Figure 2 shows the fluorescence spectra of chloroform extracts from the supernatants (A) and the aqueous fractions (B) of the same samples. Both the water and chloroform fractions had fluorescent properties, suggesting that supernatants from oxidized LG suspensions contained both hydrophilic and amphiphilic fluorophore oxidation products.

### 2.2. High-Performance Liquid Chromatography (HPLC) and Mass Spectrometry Analyses of Lipofuscin Granule BisRets and BisRet-OX

**Figure 3:**
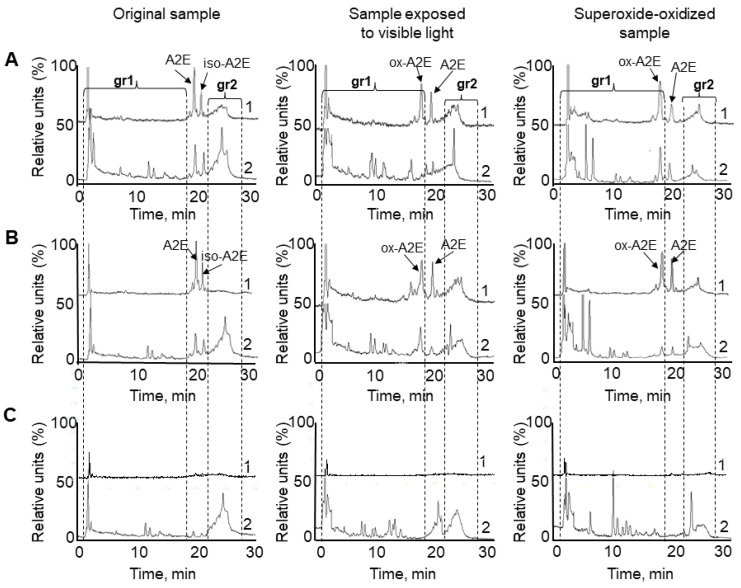
HPLC analysis of chloroform extracts from the studied samples in Figure 1. (**A**) lipofuscin granule (LG) suspension, (**B**) LG sediment, and (**C**) supernatant. Vertical dotted lines indicate groups of peaks: (gr1), group of peaks corresponding to BisRet-OX, containing mono- and bis-oxy A2E; and (gr2), group of peaks corresponding to bisretinoids and their oxidized forms. Detection at wavelengths of 430 nm (1) and 365 nm (2).

To determine the nature of supernatant compounds, we performed a comparative HPLC analysis (Figure 3) of the chloroform fractions from all samples presented in Figure 1: LG suspensions (A), LG sediments (B), and supernatants (C).

HPLC analysis of samples obtained from original non-oxidized LG suspensions revealed that almost all detectable products were present in the first group of peaks (gr1) on the chromatogram pass from LGs into the supernatant (Figure 3C, original sample). We postulate that these products could correspond to BisRet-OX [31,33]. Moreover, in the supernatants, products belonging to the second group of peaks (gr2) were present, corresponding to BisRets and their oxidized forms, identified in prior studies [32,41,42,48]. 

Importantly, the products formed after exposure of LG suspensions to photo-oxidation or superoxide absorbed primarily in the shorter wavelength range of the spectrum (365 nm), and passed into the supernatant (Figure 3C, sample exposed to visible light, and superoxide-oxidized sample). A2E, its iso-form, and ox-A2E were detected in trace amounts at both 430 and 365 nm in chloroform extracts from the supernatants of all samples (Figure 3C). This indicates that A2E and its slightly oxidized forms (ox-A2E) [33] practically do not diffuse from LGs into the aqueous medium of the supernatants.

**Figure 4:**
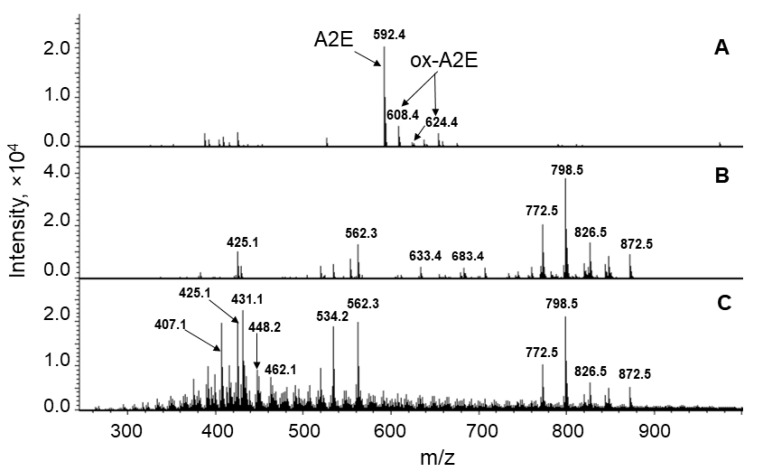
Mass spectrometry analysis of A2E and its singly and doubly oxidized forms (**A**), supernatant from the original lipofuscin granule (LG) suspension (**B**), and supernatant from LG suspension irradiated for 60 min (**C**).

Mass spectrometry analysis confirmed the HPLC data (Figure 4). A2E (*m*/*z* = 592) and its slightly oxidized forms (singly oxidized A2E *m*/*z* = 608; doubly oxidized A2E *m*/*z* = 624) [33] (Figure 4A) were nearly absent in supernatants (Figure 4B,C). This suggested that A2E and its minimally oxidized forms did not significantly diffuse through the LG membranes into the RPE cell cytoplasm.

Importantly, supernatant BisRet-Ox content increased after irradiation of the LG suspension (Figure 4C). For example, the amounts of both newly formed BisRet-OX and the contents of existing BisRet-OX in the sample increased after irradiation (Figure 4C). These BisRet-OX products had lower masses than A2E (*m*/*z* = 422, 432, 438, 448, 464, 470, 488, and 504) [38,39]. This suggested that both the oxidation and destruction of bisretinoids occurred during irradiation. 

The HPLC data thus revealed that most of the products in the chloroform extracts of LG suspensions detected at 365 nm were BisRet-OX, which have amphiphilic properties and can potentially diffuse through the LG membrane into the RPE cell cytoplasm.

### 2.3. Analysis of Thiobarbituric Acid (TBA)-Active Product Content in Lipofuscin Granule (LG) Suspensions and Supernatants 

Fluorescence spectroscopy and HPLC analyses suggested that hydrophilic and amphiphilic BisRet-OX could contain cytotoxic active carbonyl compounds, especially aldehydes and ketones. This assumption is also supported by studies that identified glyoxal formation during A2E oxidation [45].

**Figure 5:**
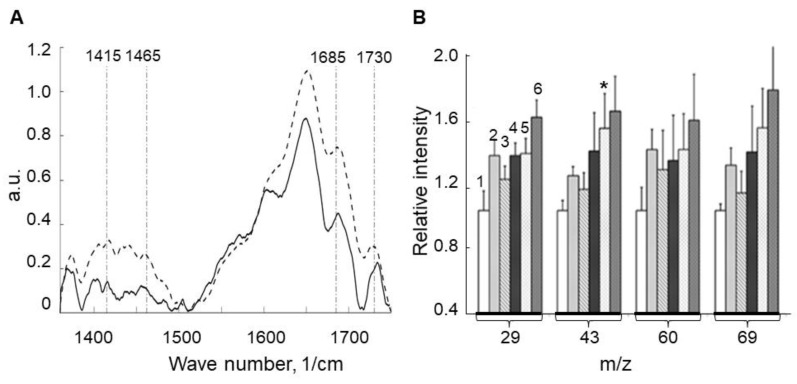
(**A**) Averaged Raman spectra for lipofuscin granule (LG) suspensions before (solid line) and after (dotted line) visible light irradiation for 100 min. Three independent experiments were conducted. In each experiment, 25 spectra were obtained (*p* < 0.05). (**B**) Time-of-flight secondary ion mass spectrometry (ToF–SIMS) analysis of LG suspensions before irradiation (1) and after visible light irradiation for 2 (2), 10 (3), 40 (4), 100 (5), and 160 (6) min. On the abscissa axis, the numbers correspond to the mass of positive fragment ions, containing carbonyl groups (29: CHO+; 43: C_2_H_3_O+; 60: C_2_H_4_O_2_+; 69: C_4_H_5_O+). On the ordinate axis, the relative intensities of the corresponding positive fragment ions are plotted as relative units. Data are presented as means ± SD from nine independent experiments. * *p* < 0.01.

We applied Raman spectroscopy analysis (BCARS) of LG suspensions before and after visible light irradiation to determine if free-state aldehydes and ketones were present in the LGs. Raman spectra identified bands of aldehydes and ketones such as ~1415, ~1465, ~1685, and ~1730 1/cm [49] (Figure 5A). After light irradiation, these bands were clearly present, including the ratio of C=O bands (1685 and 1730 1/cm) relative to aromatic C=C bands (~1600 1/cm, which we propose should not essentially change) [50], which has grown by about 1.5–2 times. Thus, exposure to light increased the contents of free-state aldehydes and ketones. 

The formation of oxygen-containing products, such as aldehydes and ketones, was also assessed by time-of-flight secondary ion mass spectrometry (ToF–SIMS) analysis of characteristic fragment ions containing carbonyl groups (*m*/*z* = 29: CHO+ ion; *m*/*z* = 43: C_2_H_3_O+ ion; *m*/*z* = 60: C_2_H_4_O_2_+ ion; *m*/*z* = 69: C_4_H_5_O+ ion). These ions accumulated during light irradiation (Figure 5B). This was consistent with prior findings [29,40]. Therefore, the BCARS and ToF–SIMS data revealed that aldehydes and ketones accumulated in LGs in the free state. 

**Figure 6:**
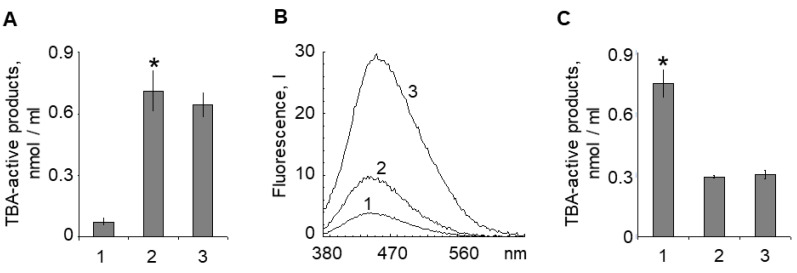
(**A**) Concentrations of thiobarbituric acid (TBA)-active products in samples of the supernatants from the original lipofuscin granule (LG) suspension (1), LG suspension irradiated with visible light for 60 min (2), or LG suspension oxidized by superoxide radicals (3). (**B**) Fluorescence spectra of the supernatants (the spectrum numbers correspond to the sample numbers in panel **A**). The fluorescence excitation wavelength was 365 nm. (**C**) Supernatant content of TBA-active products from LG suspension irradiated with visible light for 90 min (1), and in the aqueous (2) and chloroform (3) fractions. Data are presented as means ± SD from three independent experiments. * *p* < 0.05.

We used TBA-active product (reactive carbonyls) registration in the supernatants to determine the ability of aldehydes and ketones to diffuse through the LG membrane into the RPE cell cytoplasm (Figure 6). Exposure of LG BisRets to visible light or superoxide radicals resulted in significant accumulation of TBA-active products in the supernatants (Figure 6A). 

Interestingly, the fluorescence intensities of the supernatants after LG suspension irradiation with visible light or oxidation by superoxide radicals (Figure 6B, spectra 2 and 3) did not correlate with the content of TBA-active products in these samples (Figure 6A). This suggested that the composition of the oxidized products was not identical in the supernatants from the LG suspensions exposed to visible light irradiation or superoxide. This potentially suggested that not all fluorescent compounds were TBA-active products (Figure 6B). 

In addition, a comparative analysis of the hydrophilic and amphiphilic TBA-active product contents contained in the supernatant from the LG suspension irradiated with visible light was conducted. Aqueous and chloroform fractions of the supernatant were separately prepared, and the content of TBA-active products was measured. The TBA-active product contents were nearly equal in both the water-soluble and chloroform fractions of the supernatant (Figure 6C, columns 2 and 3, respectively). This suggests that the TBA-active products of photo-oxidized Bis-Ret fluorophores were likely a mixture consisting of fully oxidized, water-soluble substances and partially oxidized, lipid affinity-preserving substances.

### 2.4. BisRet-OX-Induced Protein and Lipid Modification Products 

The ability of supernatant products obtained from non-irradiated and irradiated LG suspensions to induce protein and lipid modifications in the absence of light was investigated. Photoreceptor outer segments (POSs) from bovine retinas were used as a protein and lipid substrate. The proposed modification occurs via the interaction of carbonyl products present in the supernatants with the free amino groups of proteins and lipids, especially after irradiation of the LG suspension. Fluorescent Schiff bases with emission maxima at 440–455 nm are formed through this interaction. Importantly, the LG bisretinoid fluorophores themselves could not contribute to fluorescence in this experiment, as they were separated from the modified proteins and lipids by dialysis (see Materials and Methods, Section 4.11). 

**Figure 7:**
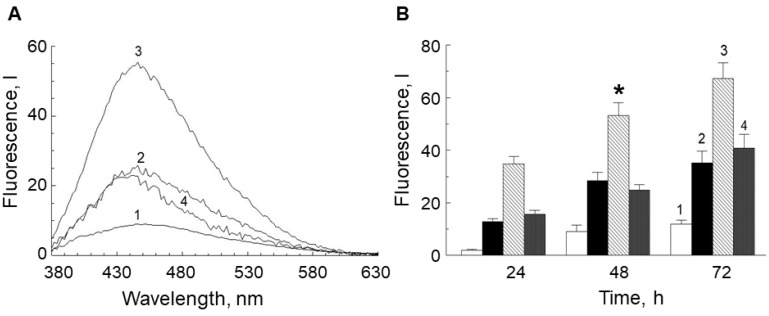
(**A**) Fluorescence spectra of photoreceptor outer segment (POS) suspensions after 48 h incubation in the dark with (1) control without additives; (2) supernatant from the original lipofuscin granule (LG) suspension; (3) supernatant from LG suspension irradiated with visible light for 60 min; and (4) supernatant from LG suspension irradiated with visible light for 60 min, and in the presence of aminoguanidine (4 mM). The fluorescence excitation wavelength was 365 nm. (**B**) Dynamics of the increase in intensity of POS suspension fluorescence after incubation in the dark for 24, 48, and 72 h. The column designations correspond to the designations of the samples on panel (**A**). Data are presented as means ± SD from three independent experiments. * *p* < 0.05.

Figure 7 demonstrates that incubation of the POS suspension with water-soluble fractions obtained from the LG suspensions significantly increased fluorescence intensity.

A noticeable increase in fluorescence was observed in the POS suspension incubated with the supernatant from the LG suspension irradiated with visible light (Figure 7A, 3). However, in the presence of the glycation inhibitor, aminoguanidine, the water-soluble fraction of the irradiated LG suspension did not significantly increase in intensity of POS fluorescence (Figure 7A, 4), and in fact had a similar effect of the water-soluble fraction of the non-irradiated LG suspension. Aminoguanidine reacts with dicarbonyl compounds, such as methylglyoxal and glyoxal, to form 3-amino-1,2,4-triazine derivatives and prevent the glycation process by these agents [51].

The dynamics of the increase in the POS fluorescence intensity for all experiments are shown in Figure 7B. The concentration of fluorescent products in POS samples incubated with the water-soluble fractions from irradiated LG suspensions (column 3) increased with incubation time. Importantly, in POS samples containing supernatant from a non-irradiated LG suspension, an increase in fluorescence intensity was also observed. This is because the unirradiated supernatant also contained carbonyl compounds, but in lower concentrations (Figure 5 and Figure 6). In addition, during prolonged incubation of POS suspensions, which contain abundant polyunsaturated fatty acid residues [52], at 37 °C, these fatty acids would be expected to undergo auto-oxidation resulting in formation of carbonyl compounds, which also induce modification of POS proteins and lipids.

## 3. Discussion

In the present study, the features of LG BisRet-OX from RPE cells were investigated. BisRet-OXs had both hydrophilic and amphiphilic properties, allowing their diffusion from the LG into the RPE cell cytoplasm. These products can be formed not only during BisRet photo-oxidation, but also during the oxidative destruction of BisRets in the dark via non-lipofuscin ROS. 

The experimental data indicate that the hydrophilic and amphiphilic BisRet-OX compounds are TBA-active. Reactive carbonyls are highly cytotoxic, and can cause carbonyl stress [53,54]. It is important to note that these carbonyl products contain water-soluble amphiphilic compounds that can damage not only the water-soluble proteins of the cytoplasm, but also various membrane structures of the cell. Since carbonyl products are long-lived, they can bind tightly with long-lived proteins, such as collagen [55] or hemoglobin [56], under physiological conditions, resulting in the formation of advanced glycation end products. Protein glycation caused by these substances can activate inflammatory processes, and thus is of great importance in the pathogenesis of many eye diseases, including AMD, in which LGs accumulate in RPE cells.

Therefore, despite the fact that the BisRet-OX species absorb in the short-wavelength region of the spectrum (UV) and cannot be photoactive in vivo, they are nevertheless toxic to RPE cells. Almost all detectable BisRet-OX species have the potential to exit LGs and enter the RPE cell cytoplasm and can thus damage cellular organelles and macromolecules. Therefore, formation of BisRet-OX-containing active carbonyls, which accumulate in the LGs of RPE cells, may play an important role in the pathogenesis of eye disease, including AMD.

## 4. Materials and Methods

### 4.1. Tissues and Reagents

Experiments on human cadaver eye tissues were performed in compliance with officially accepted procedures, specifically the Russian Federation law N 4180-I dated 22 December 1992, “On human organs or tissue transplantation” (with the most recent modifications and additions dated 8 December 2020). Human cadaver eyes without ophthalmologic disease were obtained from the Eye Tissue Bank of the S.N. Fyodorov Eye Microsurgery Complex (Moscow, Russia) within 10 h of donor death. Donor age ranged from 50–75 years. Collection was conducted in accordance with local ethics requirements, as described in detail previously [31]. Permission was obtained from the chief medical officer of the S.N. Fyodorov Eye Microsurgery Complex, under a scientific collaboration agreement between the Complex and the Emanuel Institute of Biochemical Physics dated 11 January, 2011 to perform scientific research in the Laboratory of Physical and Chemical Bases of Vision at the Institute with RPE from cadaver eyes. 

All reagents were purchased from Sigma–Aldrich (St. Louis, MO, USA) and Fluka (Buchs, Switzerland).

Fresh bovine eyes (Bos taurus) were purchased from a meat processing factory.

### 4.2. Isolation of Lipofuscin Granules (LGs) 

LGs were isolated from the RPE of 40 cadaver eyes as described previously [6]. The initial LG concentration was 5 × 10^8^ granules/mL. Water-soluble fractions from LG suspension were obtained as follows. For experiments, LGs were resuspended in 0.1 M potassium phosphate buffer (pH 7.3) to the concentration of 2.0 × 10^8^ granules/mL. Then suspension was divided into two samples of equal volume. The experimental sample was irradiated for 30–160 min (depending on the experiment) with visible light (400–700 nm) of a KGM 24–150 lamp (80 mW/cm^2^) at room temperature and constant stirring. The control sample remained all this time in complete darkness. Thereafter, the samples were centrifuged at 15,000 ×*g* in a Beckman Allegra 64R centrifuge for 15 min, and the obtained supernatants were used in the experiments. The sediments containing LGs were homogenized in 1 mL 0.1 M potassium phosphate buffer. 

All stages of sample preparation were conducted under subdued lighting.

### 4.3. Preparation of Photoreceptor Outer Segments (POSs) Containing Rhodopsin 

POSs from bovine retina tissue, which was isolated within 3 h after slaughter, were prepared as described previously [57]. Retinas were added to 50% sucrose solution in Buffer A comprising 10 mM MOPS pH 7.4, 30 mM NaCl, 60 mM KCl, 2 mM MgCl_2_, 0.1 mM phenylmethylsulfonyl fluoride (PMSF), 1 mM dithiothreitol, and 0.01% NaN_3_ (1 mL per each retina). The suspension was vigorously shaken for 3 min and centrifuged at 2000× *g* for 40 min at 4 °C. The supernatant was diluted 2.5-fold with Buffer A and centrifuged at 2000× *g* for 60 min at 4 °C. The pellet was resuspended in 25 mL of 40% sucrose in Buffer A, and 10 mL of Buffer A was layered on top following centrifugation at 25,000× *g* for 60 min at 4 °C on a Beckman Coulter Avanti J30I centrifuge equipped with a JS 24.38 rotor. The POS fraction was sampled at the buffer-sucrose interface and diluted with Buffer A to a density of 1.05 g/cm^3^. POSs were then sedimented by centrifugation at 41,400× *g* for 30 min at 4 °C on a Beckman Coulter Allegra 64R centrifuge equipped with a F0650 rotor and resuspended with the required amount of 0.1 M phosphate buffer, pH 7.4. All manipulation of samples containing rhodopsin was carried out under dim red illumination.

### 4.4. High-Performance Liquid Chromatography (HPLC)

HPLC analysis was conducted using a Knauer chromatograph system (Knauers, Berlin, Germany) equipped with a Diaspher-120-C18 (4 × 250 mm; sorbent size, 5 μm) column, as described previously [58]. HPLC analysis (K-2501 detector, Knauer) was performed at the wavelengths of 365 and 430 nm. 

For chromatographic analysis, chloroform extracts from LG suspension or supernatant samples were prepared using the Folch method [59], followed by drying and subsequently resuspending in 200 μL methanol, as described previously [58]. Precision for each sample was determined from two separately measured chromatograms for each individual sample at each wavelength.

### 4.5. Sample Oxidation

For photo-oxidative destruction, the LG suspension in phosphate buffer or A2E in acetonitrile were irradiated at room temperature under constant stirring using a 150 W incandescent lamp with a heat filter (KGM 24–150, 400–700 nm). The luminous flux density irradiating the sample was 80 mWm−2 for visible light (400–700 nm), as determined by a photometer (Spectra-Physics 407A, Milpitas, CA, USA).

For oxidative destruction, reaction of the samples with superoxide radicals was conducted in the dark by adding an equal mass of dry potassium peroxide (KO_2_) to each sample (10–25 mg). Sample incubation with superoxide was conducted for 1 h with periodic stirring at room temperature.

### 4.6. A2E Synthesis

A2E was prepared from all-trans retinal and ethanolamine in acetic acid and ethanol, as described previously [60]. A2E and its photo-oxidation products were identified using a 7T LTQ FT mass spectrometer (Thermo Electron Corp., Bremen, Germany) equipped with an electrospray ion source, as described previously [32]. To reliably identify A2E, we performed MC/MC studies in a linear quadrupole trap, in which molecular ions with a certain mass-to-charge ratio (M/Z) were isolated from other ionization products and subjected to collision-induced dissociation (ESI-CID) with subsequent mass spectrometry analysis of the fragmentation products [39]. Mass spectra were processed and analyzed using Qual Browser 1.4 software.

A2E purity was monitored by HPLC (see Section 2.3). A2E concentration was determined spectrally using a Shimadzu UV-1700 spectrophotometer (Shimadzu, Kyoto, Japan) at a wavelength of 430 nm with *ε* = 3.1 × 10^4^ M^−1^ cm^−1^.

### 4.7. Fluorescence Spectroscopy 

Fluorescence spectra were recorded using an RF-5301 PC fluorometer (Shimadzu, Kyoto, Japan) equipped with an R955 photomultiplier tube detector (Hamamatsu Photonics K.K., Hamamatsu City, Shizuoka Pref.430-8587, Japan). Following excitation at 340, 365, or 488 nm, emission spectra were obtained in the regions of 360–660, 380–660, and 500–700 nm, respectively, at sampling intervals of 1 nm. 

### 4.8. Mass Spectrometry Analysis

Sample mass spectra were obtained using a qTOF mass spectrometer MaXis 4G (Bruker Daltonics, Bremen, Germany) with an electrospray ion source (ESI) in positive mode. ESI source parameters were the following: capillary 3.4 kV, nebulizing gas pressure 0.7 bar, drying gas flow rate 6 L/min, and drying gas temperature 200 °C. Samples were dissolved into a mixture of acetonitrile and water (1:1) and sprayed at a flow rate of 3 µL/min. Measurements were conducted in the range of *m*/*z* 100–1500. Mass spectra were processed and analyzed using data analysis software (Bruker Daltonics, Bremen, Germany).

To detect BisRet-OXs, such as aldehydes and ketones, dried LG samples were analyzed by time-of-flight secondary ion mass spectrometry (ToF–SIMS) (ION-ToF, Münster, Germany) using 30 keV Bi_3_^+^ primary ions. The analysis area was 300 × 300 μm^2^ with a primary ion dose density of ~4 × 10^11^ ions/cm^2^. For each sample, at least nine such areas were analyzed. All spectra were recorded in both positive and negative ion modes. An electron flood gun was activated to avoid the charging effect during analysis. Mass spectra were processed and analyzed by SurfaceLab software (ION-TOF, Münster, Germany). Ion yields were calculated as the number of ions with specific masses divided by total ion counts.

### 4.9. Raman Spectroscopy Analysis

Raman spectroscopy analysis was conducted using a Broadband coherent anti-Stokes Raman scattering (BCARS) microspectrometer implemented as part of a femtosecond laser complex [61] representing a two-pulse collinear scheme for CARS generation [62]. Related spectra were transformed to Raman bands using the CARS-variation of MEM [63,64]. LG sample preparation and experimental conditions have been described in detail in prior studies [50].

### 4.10. Determination of Carbonyls 

The relative toxicities of products that accumulated from oxidative and photo-oxidative degradation of BisRets in LG suspensions, LG sediments, and supernatants were assessed by the content of carbonyls reacting with TBA-active products [65]. The content of TBA-active products in chloroform extracts was determined after vacuum pump evaporation of the chloroform (Vacuubrand MZ2C NT+AK+M+D, Wertheim, Germany), followed by sediment solubilization in 0.1 M potassium phosphate buffer (pH 7.3). TBA-active product concentration was determined spectrally in photo-oxidized and superoxide-oxidized LG samples at 532 nm [66] using a Shimadzu UV-1700 spectrophotometer (Shimadzu, Kyoto, Japan). The original non-oxidized granules were used as controls.

### 4.11. Determination of Protein and Lipid Modification Products 

Protein and lipid modifications were assessed by the formation of fluorescent Schiff bases in the reaction of aldehydes with the free amino groups of proteins and lipids. POS suspensions were used as a protein and lipid substrate (see Section 4.3). The incubation medium contained 0.1 M sterile potassium phosphate buffer, pH 7.4; 0.3–0.6 mg/mL POS protein, 3–4 mM sodium azide, and 0.4–0.7 mL supernatants from irradiated and non-irradiated (control) LG suspensions. As additional control samples, we used samples containing only POS suspensions without supernatants, supernatants without added proteins, and samples containing a glycation inhibitor, aminoguanidine (4 mM). The samples were incubated at 37 °C in the dark with constant stirring for 24, 48, or 72 h. 

After incubation, sample aliquots were dialyzed against phosphate buffer to remove unreacted low molecular weight molecules. For dialysis we used a Float-A-Lyser cellulose-ether membrane (SPECTRUM Labs, New Brunswick, NJ, USA), which is permeable to molecules <3.5 kDa. Dialysis was conducted for 25 h at 6 °C. After dialysis, the fluorescence spectra of the modified proteins and lipids were measured at an excitation wavelength of 365 nm. The modified protein and lipid content was estimated by the magnitude of the fluorescence amplitude, measured at the emission maximum of 440–455 nm.

### 4.12. Statistical Analysis

Statistical significance of experimental results was analyzed using a Student’s *t*-test. Results with *p* < 0.05 were considered statistically significant. Data are presented as mean ± standard deviation (SD). 

## Data Availability

The data presented in this study are available in the article and on request from the corresponding author.

## References

[1] Nivison-Smith L., Milston R., Madigan M., Kalloniatis M. (2014). Age-related macular degeneration: Linking clinical presentation to pathology. Optom. Vis. Sci..

[2] Fisher C.R., Ferrington D.A. (2018). Perspective on AMD pathobiology: A bioenergetic crisis in the RPE. Investig. Ophthalmol. Vis. Sci..

[3] Abokyi S., To C.-H., Lam T.T., Tse D.Y. (2020). Central role of oxidative stress in age-related macular degeneration: Evidence from a review of the molecular mechanisms and animal models. Oxidative Med. Cell. Longev..

[4] Beatty S., Koh H.-H., Phil M., Henson D., Boulton M. (2000). The Role of Oxidative Stress in the Pathogenesis of Age-Related Macular Degeneration. Surv. Ophthalmol..

[5] Kohen R., Nyska A. (2002). Invited Review: Oxidation of biological systems: Oxidative stress phenomena, antioxidants, redox reactions, and methods for their quantification. Toxicol. Pathol..

[6] Boulton M., Dontsov A., Jarvis-Evans J., Ostrovsky M., Svistunenko D. (1993). Lipofuscin is a photoinducible free radical generator. J. Photochem. Photobiol. B Biol..

[7] Ostrovsky M.A., Dontsov A.E., Sakina N.L., Boulton M., Jarvis-Evans J. (1992). The ability of lipofuscin granules of the retinal pigment epithelium of the human eye a photosensitized peroxidation lipid by the action of visible light. Sens. Syst..

[8] Kennedy C.J., Rakoczy P.E., Constable I.J. (1995). Lipofuscin of the retinal pigment epithelium: A review. Eye.

[9] Feldman T.B., Yakovleva M.A., Larichev A.V., Arbukhanova P.M., Radchenko A.S., Borzenok S.A., Kuzmin V.A., Ostrovsky M.A. (2018). Spectral analysis of fundus autofluorescence pattern as a tool to detect early stages of degeneration in the retina and retinal pigment epithelium. Eye.

[10] Holz F.G., Schmitz-Valckenberg S., Fleckenstein M. (2014). Recent developments in the treatment of age-related macular degeneration. J. Clin. Investig..

[11] Shaw P.X., Stiles T., Douglas C., Ho D., Fan W., Du H., Xiao X. (2016). Oxidative stress, innate immunity, and age-related macular degeneration. AIMS Mol. Sci..

[12] Tomany S.C., Cruickshanks K.J., Klein R., Klein B.E., Knudtson M.D. (2004). Sunlight and the 10-year incidence of age-related maculopathy: The Beaver Dam Eye Study. Arch. Ophthalmol..

[13] Ueda K., Kim H.J., Zhao J., Song Y., Dunaief J.L., Sparrow J.R. (2018). Bisretinoid photodegradation is likely not a good thing. Adv. Exp. Med. Biol..

[14] Feeney-Burns L., Hilderbrand E.S., Eldridge S. (1984). Aging human RPE: Morphometric analysis of macular, equatorial, and peripheral cells. Investig. Ophthalmol. Vis. Sci..

[15] Jung T., Bader N., Grune T. (2007). Lipofuscin. Formation, distribution, and metabolic consequences. Ann. NY Acad. Sci..

[16] Yin D. (1996). Biochemical basis of lipofuscin, ceroid, and age pigment-like fluorophores. Free Rad Biol. Med..

[17] Holz F.G., Pauleikhoff D., Klein R., Bird A.C. (2004). Pathogenesis of lesions in late age-related macular disease. Am. J. Ophthalmol..

[18] Katz M.L. (2002). Potential role of retinal pigment epithelial lipofuscin accumulation in age-related macular degeneration. Arch. Gerontol. Geriatr..

[19] Sparrow J.R., Boulton M.E. (2005). RPE lipofuscin and its role in retinal pathobiology. Exp. Eye Res..

[20] Feeney-Burns L., Eldred G.E. (1983). The fate of the phagosome: Conversion to ‘age-pigment’ and impact in human retinal pigment epithelium. Trans. Ophthalmol. Soc. UK.

[21] Holz F.G., Schütt F., Kopitz J., Eldred G.E., Kruse F.E., Völcker H.E., Cantz M. (1999). Inhibition of lysosomal degradative functions in RPE cells by a retinoid component of lipofuscin. Investig. Ophthalmol. Vis. Sci..

[22] Adler IV L., Chen C., Koutalos Y. (2017). All-trans retinal levels and formation of lipofuscin precursors after bleaching in rod photoreceptors from wild type and Abca4 -/- mice. Exp. Eye Res..

[23] Lamb L.E., Simon J.D. (2007). A2E: A Component of ocular lipofuscin. Photochem. Photobiol..

[24] Sakai N., Decatur J., Nakanishi K., Eldred G.E. (1996). Ocular age pigment “A2E”: An unprecedented pyridinium bisretinoid. J. Am. Chem. Soc..

[25] Sparrow J.R., Kim S.R., Cuervo A.M., Bandhyopadhyayand U. (2008). A2E, A Pigment of RPE Lipofuscin, is Generated from the Precursor, A2PE by a lysosomal enzyme activity. Adv. Exp. Med. Biol..

[26] Dontsov A.E., Sakina N.L., Bilinska B., Krzyzanowski L., Feldman T.B., Ostrovsky M.A. (2005). Comparison of the photosensitizing effect of lipofuscin granules from the pigment epithelium of the human eye and their fluorophore A2E. Dokl. Biochem. Biophys..

[27] Rozanowka M., Sarna T. (2005). Light-induced damage to the retina: Role of rhodopsin chromophore revisited. Photochem. Photobiol..

[28] Scimone C., Alibrandi S., Scalinci S.Z., Battagliola E.T., D’Angelo R., Sidoti A., Donato L. (2020). Expression of pro-angiogenic markers is enhanced by blue light in human RPE cells. Antioxidants.

[29] Wu Y., Yanase E., Feng X., Siegel M.M., Sparrow J.R. (2010). Structural characterization of bisretinoid A2E photocleavage products and implications for age-related macular degeneration. Proc. Natl. Acad. Sci. USA.

[30] Ben-Shabat S., Itagaki Y., Jockusch S., Sparrow J.R., Turro N.J., Nakanishi K. (2002). Formation of a nona-oxirane from A2E, a lipofuscin fluorophore related to macular degeneration, and evidence of singlet oxygen involvement. Angew. Chem. Int. Ed. Engl..

[31] Feldman T.B., Yakovleva M.A., Arbukhanova P.M., Borzenok S.A., Kononikhin A.S., Popov I.A., Nikolaev E.N., Ostrovsky M.A. (2015). Changes in spectral properties and composition of lipofuscin fluorophores from human retinal pigment epithelium with age and pathology. Anal. Bioanal. Chem..

[32] Sparrow J.R., Gregory-Roberts E., Yamamoto K., Blonska A., Ghosh S.K., Ueda K., Zhou J. (2012). The bisretinoids of retinal pigment epithelium. Prog. Retin. Eye Res..

[33] Yakovleva M.A., Sakina N.L., Kononikhin A.S., Feldman T.B., Nikolaev E.N., Dontsov A.E., Ostrovsky M.A. (2006). Detection and study of the products of photooxidation of N-Retinylidene-N-retinylethanolamine (A2E), the fluorophore of lipofuscin granules from retinal pigment epithelium of human donor eyes. Dokl. Biochem. Biophys..

[34] Dontsov A.E., Sakina N.L., Golubkov A.M., Ostrovsky M.A. (2009). Light-induced release of A2E photooxidation toxic products from lipofuscin granules of human retinal pigment epithelium. Dokl. Biochem. Biophys..

[35] Yoon K.D., Yamamoto K., Ueda K., Zhou J., Sparrow J.R. (2012). A novel source of methylglyoxal and glyoxal in retina: Implications for age-related macular degeneration. PLoS ONE.

[36] Zorov D.B., Juhaszova M., Sollott S.J. (2014). Mitochondrial reactive oxygen species (ROS) and ROS-induced ROS release. Phys. Rev..

[37] Donato L., Scimone C., Alibrandi S., Pitruzzella A., Scalia F., D’Angelo R., Sidoti A. (2020). Possible A2E mutagenic effects on RPE mitochondrial DNA from innovative RNA-Seq bioinformatics pipeline. Antioxidants.

[38] Schütt F., Davies S., Kopitz J., Boulton M., Holz F.G. (2000). A retinoid constituent of lipofuscin, A2E is a photosensitizer in human retinal pigment epithelial cells. Ophthalmologe.

[39] Sparrow J.R., Nakanishi K., Parish C.A. (2000). The lipofuscin fluorophore A2E mediates blue light-induced damage in retinal pigmented epithelial cells. Investig. Ophthalmol. Vis. Sci..

[40] Wang Z., Keller L.M.M., Dillon J., Gaillard E.R. (2006). Oxidation of A2E results in the formation of highly reactive aldehydes and ketones. Photochem. Photobiol..

[41] Murdaugh L.S., Avalle L.B., Mandal S., Dill A.E., Dillon J., Simon J.D., Gaillard E.R. (2010). Compositional studies of human RPE lipofuscin. J. Mass Spectrom..

[42] Murdaugh L.S., Mandal S., Dill A.E., Dillon J., Simon J.D., Gaillard E.R. (2011). Compositional studies of human RPE lipofuscin: Mechanisms of molecular modifications. J. Mass Spectrom..

[43] Dillon J., Wang Z., Avalle L.B., Gaillard E.R. (2004). The photochemical oxidation of A2E results in the formation of a 5,8,5’,8’-bis-furanoid oxide. Exp. Eye Res..

[44] Jang Y.P., Matsuda H., Itagaki Y., Nakanishi K., Sparrow J.R. (2005). Characterization of peroxy-A2E and furan-A2E photooxidation products and detection in human and mouse retinal pigment epithelial cell lipofuscin. J. Biol. Chem..

[45] Sparrow J.R., Vollmer-Snarr H.R., Zhou J., Jang Y.P., Jockusch S., Itagaki Y., Nakanishi K. (2003). A2E-epoxides damage DNA in retinal pigment epithelial cells. Vitamin E and other antioxidants inhibit A2E-epoxide formation. J. Biol. Chem..

[46] Feldman T.B., Yakovleva M.A., Dontsov A.E., Ostrovsky M.A. (2010). Fluorescence emission and excitation spectra of fluorophores of lipofuscin granules isolated from retinal pigment epithelium of human cadaver eyes. Russ. Chem. Bull. Int. Ed..

[47] Holz F.G., Schmitz-Valckenberg S., Spaide R.F., Bird A.C. (2007). Atlas of Fundus Autofluorescence Imaging.

[48] Kim S.R., Jang Y.P., Sparrow J.R. (2010). Photooxidation of RPE lipofuscin bisretinoids enhances fluorescence intensity. Vis. Res..

[49] Smith B.C. (2017). The carbonyl group, Part I: Introduction. Spectroscopy.

[50] Aybush A.V., Gulin A.A., Vasin A.A., Dontsov A.E., Nadtochenko V.A., Ostrovsky M.A. (2020). Multimodal approach to reveal the effect of light irradiation on chemical composition of lipofuscin granules of human RPE tissues. J. Phys. Conf. Ser..

[51] Thornalley P.J. (2003). Use of aminoguanidine to prevent the formation of advanced glycation end products. Arch. Biochem. Biophys..

[52] Bazan H.E., Bazan N.G., Feeney-Burns L., Berman E.R. (1990). Lipids in human lipofuscin-enriched subcellular fractions of two age populations. Comparison with rod outer segments and neural retina. Investig. Ophthalmol. Vis. Sci..

[53] Ergin V., Ebrahimi R., Karasu C. (2013). Carbonyl stress in aging process: Role of vitamins and phytochemicals as redox regulators. Aging. Dis..

[54] Schleicher E.D., Bierhaus A., Haring H.U., Nawroth P.P., Lehmann R. (2001). Chemistry and pathobiology of advanced glycation end products. Contrib. Nephrol..

[55] Munch G., Schicktanz D., Behme A., Gerlach M., Riederer P., Palm D., Schinzel R. (1999). Amino acid specificity of glycation and protein-AGE crosslinking reactivities determined with a dipeptide SPOT library. Nat. Biotechnol..

[56] Ott C., Jacobs K., Haucke E., Navarrete Santos A., Grune T., Simm A. (2014). Role of advanced glycation end products in cellular signaling. Redox. Biol..

[57] Feldman T.B., Ivankov O.I., Kuklin A.I., Murugova T.N., Yakovleva M.A., Smitienko O.A., Kolchugina I.B., Round A., Gordeliy V.I., Belushkin A.V. (2019). Small-angle neutron and X-ray scattering analysis of the supramolecular organization of rhodopsin in photoreceptor membrane. Biochim. Biophys. Acta (BBA) Biomembranes.

[58] Yakovleva M.A., Radchenko A.S., Feldman T.B., Kostyukov A.A., Arbukhanova P.M., Borzenok S.A., Kuzmin V.A., Ostrovsky M.A. (2020). Fluorescence characteristics of lipofuscin fluorophores from human retinal pigment epithelium. Photochem. Photobiol. Sci..

[59] Folch J., Lees M., Sloane Stanley G.H. (1957). A simple method for the isolation and purification of total lipids from animal tissues. J. Biol. Chem..

[60] Parish C.A., Hashimoto M., Nakanishi K., Dillon J., Sparrow J. (1998). Isolation and one-step preparation of A2E and iso-A2E, fluorophores from human retinal pigment epithelium. Proc. Natl. Acad. Sci. USA.

[61] Nadtochenko V., Denisov N., Aybush A., Gostev F., Shelaev I., Titov A., Umanskiy S., Cherepanov D. (2017). Ultrafast spectroscopy of fano-like resonance between optical phonon and excitons in CdSe quantum dots: Dependence of coherent vibrational wave-packet dynamics on pump fluence. Nanomaterials.

[62] Zhang C., Zhang D., Cheng J. (2015). Coherent Raman scattering microscopy in biology and medicine. Ann. Rev. Biomed. Eng..

[63] Okuno M., Kano H., Leproux P., Couderc V., Day J.P.R., Bonn M., Hamaguchi H. (2010). Quantitative CARS molecular fingerprinting of single living cells with the use of the maximum entropy method. Angew. Chem. Int. Ed..

[64] Vartiainen E., Rinia H., Muller M., Bonn M. (2006). Direct extraction of Raman line-shapes from congested CARS spectra. Opt. Express.

[65] Esterbauer H., Cheeseman K.H. (1990). Determination of aldehydic lipid peroxidation products: Malonaldehyde and 4-hydroxynonenal. Methods Enzym..

[66] Buege J.A., Aust S.D. (1978). Microsomal lipid peroxidation. Methods Enzym..

